# Prevalence of suicidal behaviours and its correlates in latvian general population: 2019-2020

**DOI:** 10.1192/j.eurpsy.2021.1578

**Published:** 2021-08-13

**Authors:** V.V. Vinogradova, A. Kivite-Urtane, J. Vrublevska, E. Rancans

**Affiliations:** 1 Department Of Psychiatry And Narcology, Riga Stradins University, Riga, Latvia; 2 Department Of Public Health And Epidemiology, Riga Stradins University, Riga, Latvia

**Keywords:** suicidal behaviours, General population, prevalence

## Abstract

**Introduction:**

Suicide is a challanging problem for a global public health and Latvia remains in the list of European countries with the highest rates of suicide deaths. Information about the epidemiology of suicidal behavior is required for suicide prevention strategy development.

**Objectives:**

To determine the prevalence of suicidal behavior (suicidal ideation, plan, and attempts) and associated factors in Latvian general population.

**Methods:**

Computer assisted face-to-face interviews were carried out between November 2019 and March 2020 to gather information on a representative sample of the Latvian adult population (n=2687). The study sample was selected using a stratified random sampling method. The Mini-International Neuropsychiatric Interview (MINI; version 7.0.2) was used to assess suicidality. Multinomial logistic regression was applied.

**Results:**

There were 1238 males (46.1%) and 1449 females (53.9%) recruited. Mean age of respondents was 49.9 (SD 18.2). According to the MINI, 10.6% (n=285) of respondents reported at least some level of suicidal behaviour during the last month before interview and 7.1% (n=191) had shown current suicidal behaviour at the moment of interview, 4.0% (n=108) of respondents reported about previous suicide attempts. Non-cohabitation status, unfinished primary education and economical inactivity were statistically significant associated factors for suicidal behaviour among men, but only lower level of education was for women.

**Conclusions:**

Comprehensive national suicide prevention strategy is required for reducing suicidality in Latvia. Special attention should be paid to women with lower education, and economically inactive, unmarried or non-cohabitant men, as well as man with unfinished primary education.

